# Incidence, Mortality and Positive Predictive Value of Type 1 Cardiorenal Syndrome in Acute Coronary Syndrome

**DOI:** 10.1371/journal.pone.0167166

**Published:** 2016-12-01

**Authors:** Raquel Pimienta González, Patricia Couto Comba, Marcos Rodríguez Esteban, José Juan Alemán Sánchez, Julio Hernández Afonso, María del Cristo Rodríguez Pérez, Itahisa Marcelino Rodríguez, Buenaventura Brito Díaz, Roberto Elosua, Antonio Cabrera de León

**Affiliations:** 1 Servicio de Cardiología. Hospital Universitario Nuestra Señora de la Candelaria, Santa Cruz de Tenerife, Spain; 2 Unidad de Investigación de Atención Primaria y del Hospital Universitario Nuestra Señora de la Candelaria, Santa Cruz de Tenerife, Spain; 3 Red de Investigación Cardiovascular, Instituto de Salud Carlos III, Madrid, Spain; 4 Epidemiología Cardiovascular y Genética, IMIM. Barcelona, Spain; 5 Área de Medicina Preventiva y Salud Pública, Universidad de La Laguna, La Laguna, Spain; Cincinnati Children's Hospital Medical Center, UNITED STATES

## Abstract

**Objectives:**

To determine whether the risk of cardiovascular mortality associated with cardiorenal syndrome subtype 1 (CRS1) in patients who were hospitalized for acute coronary syndrome (ACS) was greater than the expected risk based on the sum of its components, to estimate the predictive value of CRS1, and to determine whether the severity of CRS1 worsens the prognosis.

**Methods:**

Follow-up study of 1912 incident cases of ACS for 1 year after discharge. Cox regression models were estimated with time to event (in-hospital death, and readmission or death during the first year after discharge) as the dependent variable.

**Results:**

The incidence of CRS1 was 9.2/1000 person-days of hospitalization (95% CI = 8.1–10.5), but these patients accounted for 56.6% (95% CI = 47.4–65.) of all mortality. The positive predictive value of CRS1 was 29.6% (95% CI = 23.9–36.0) for in-hospital death, and 51.4% (95% CI = 44.8–58.0) for readmission or death after discharge. The risk of in-hospital death from CRS1 (RR = 18.3; 95% CI = 6.3–53.2) was greater than the sum of risks associated with either acute heart failure (RR = 7.6; 95% CI = 1.8–31.8) or acute kidney injury (RR = 2.8; 95% CI = 0.9–8.8). The risk of events associated with CRS1 also increased with syndrome severity, reaching a RR of 10.6 (95% CI = 6.2–18.1) for in-hospital death at the highest severity level.

**Conclusions:**

The effect of CRS1 on in-hospital mortality is greater than the sum of the effects associated with each of its components, and it increases with the severity of the syndrome. CRS1 accounted for more than half of all mortality, and its positive predictive value approached 30% in-hospital and 50% after discharge.

## Introduction

The simultaneous appearance of heart failure and kidney injury has been termed cardiorenal syndrome (CRS) [1–3]. According to the proposed classification, CRS is divided into five subtypes depending on the organ that initiates the cascade and leads to failure of the other organ, and on whether organ failure is acute or chronic.^1^ In CRS subtype 1 (CRS1), acute heart failure (AHF) leads to acute kidney injury (AKI) [4]. The incidence of CRS1 varies widely among studies because of differences in the definitions used and heterogeneity in the populations studied to date [5]. It has been estimated that CRS1 occurs in approximately 25%-33% of patients admitted with acute decompensated heart failure [6].

Coronary heart disease is still the cause of more than two-thirds of all heart disease-related deaths in western societies [7], and patients who develop AKI after a coronary event have significantly higher morbidity and mortality, longer hospitalization and a greater incidence of readmission [8]. In patients with acute coronary syndrome (ACS) CRS worsens the prognosis [9]. and it is not surprising that those who develop both AHF and AKI have worse outcomes than those with just one. Clinical guidelines have classically treated cardiac and renal failure separately, but it is important to elucidate the characteristics of CRS in more detail in order to enhance the integrative clinical management of this syndrome [1]. Therefore, a better awareness of the importance of CRS1 in patients with ACS is needed, and we also need to know more about whether the risk of mortality associated with CRS1 is greater than the expected risk based on the sum of its components (AHF and AKI), and whether the severity of CRS1 matters.

The present study was designed to determine whether the risk of cardiovascular mortality associated with CRS1 in patients who were hospitalized for ACS was greater than the expected risk based on the sum of its components, to estimate the positive predictive value of CRS1, and to determine whether the severity of CRS1 worsens the prognosis.

## Patients and Methods

This follow-up study was based on a cohort of incident cases of ACS in the general population. Between 1 January 2007 and 31 December 2010, we prospectively recruited all patients admitted for ACS at Nuestra Señora de Candelaria University Hospital, a tertiary reference center which serves a population of 600,000 inhabitants on the island of Tenerife (Canary Islands, Spain). The patients consented to take part of the study and provided written informed consent; and they were initially followed during their hospital stay, and then for 1 year after discharge. After discharge, patients were followed at their cardiology outpatient department appointments and primary care appointments. To avoid missing data, they were also followed by telephone interview. The sample size far exceeded the requirements for an alpha error of 1% and a beta error of 5%, assuming a ratio of 8 for unexposed/exposed to CRS in the sample. The study was approved by the hospital’s clinical research ethics committee.

The diagnosis of ACS was based on the presence of as at least 2 of the following criteria: 1) compatible clinical presentation; 2) dynamic changes in the ST segment, inverted T wave or the appearance of a new Q wave; 3) troponin I elevation above the upper limit of normality (0.6 ng/mL); 4) angiographic finding of a compatible coronary lesion.

### Heart failure and kidney function: Cardiorenal syndrome definition and categorization

The Killip–Kimball classification was used to identify heart failure during the episode, and heart failure was recorded if the patient was considered class II or higher. Use of balloon dilatation or amines depended on the stage of evolution of ACS in each patient in Killip stage III and IV, but these treatments were not included in the present analysis since they have not been shown to modify survival [10].Chronic kidney disease (CKD) was recorded when diminished creatinine clearance was documented according to an estimated glomerular filtration rate <60 mL/min in the year prior to admission; patients on dialysis were specifically excluded from the analysis. AKI was defined, according to Acute Kidney Injury Network criteria, as an acute increase in serum creatinine on admission ≥0.3 mg/dL or 1.5-fold higher than normal [11]. CRS1 was defined as the sum of the two components: AHF followed by AKI [1,3]. Treatment of acute kidney failure in patients with heart failure ranged from hydration and avoiding potentially nephrotoxic medications to dialysis in the intensive care unit, in consultation with the nephrology department.

To assess the importance of CRS severity we empirically categorized patients in 4 grades according to Killip–Kimball score and the increase in serum creatinine on admission. The combination that performed best in predicting mortality (creatinine elevations ≥0.5) was used for t study. The four categories were: 1: Killip–Kimball = II, and increase in creatinine <0.5 mg/dL (n = 29); 2: Killip–Kimball = II, but increase in serum creatinine ≥0.5 mg/dL (n = 41); 3: Killip–Kimball > II, but increase in serum creatinine <0.5 mg/dL (n = 36); 4: Killip–Kimball > II, and increase in serum creatinine ≥0.5 mg/dL (n = 110. We accepted the fourth grade as equivalent to cardiogenic shock.

### Other variables

Approximately 30% of the patients who had infarction with elevated ST segment received therapy with fibrinolytics. All these patients subsequently underwent coronary angiography and revascularization, if needed. Revascularization was done percutaneously or surgically according to the angiographic findings, and was classified as: none, surgical or percutaneous catheterization. Left ventricular dysfunction (LVD) was recorded when ejection fraction calculated from echocardiographic studies on admission was 40% or lower. Peripheral vascular disease (PVD) was diagnosed when confirmation was obtained from imaging studies for arterial disease in the lower extremities, abdominal aorta or carotid arteries, or when the patient had prior evidence of PVD (amputation or revascularization). Previous coronary artery disease was recorded when a diagnosis of angina or myocardial infarction was documented.

Antecedents of hypertension, diabetes mellitus, dyslipidemia, chronic obstructive pulmonary disease (COPD), smoking (non-smoker, former smoker or smoker) and alcohol consumption were recorded for all patients. A diagnosis of hypertension was noted when the patients were taking antihypertensive medication or if their blood pressure in hospital was ≥140/90 mmHg at least twice, and diabetes mellitus was recorded when the patients were receiving antidiabetic treatment or if their fasting serum glucose was >125 mg/dL at least twice. Dyslipidemia was noted if the patient was using statins or if serum cholesterol was >240 mg/dL. Anemia was defined as a plasma hemoglobin concentration <13 g/dL in men or <12 g/dL in women. Patients were considered smokers if they stated smoking at least 1 cigarette per day, and ex-smokers if they stated having quit at least 1 year previously. Excessive drinking was defined as alcohol consumption >40 g/day in men or >30 g/day in women. Obesity was defined as a body mass index ≥30.

### Statistical analysis

Categorical variables are summarized as relative frequencies and 95% confidence intervals (95% CI), and continuous variables as the mean ± standard deviation or the median (P_25_–P_75_). We used ANOVA tests to find associations between continuous and categorical variables, and univariate Cox models for the variables time to death or time to readmission. For associations between categorical variables we used the Pearson chi-squared test. Incidence density was calculated as the quotient of the number of events divided by the sum of hospital stays, in days, for all patients (sum of days after discharge when the event studied was death or readmission). Incidence densities, comparisons of rates (rates ratios, 95% CI, p) and positive predictive values were calculated with the application available at http://www.openepi.com.

For the multivariate analysis we adjusted two Cox regression models (A and B) with time to event as the dependent variable in a backwards stepwise procedure (Wald), with exit criteria = 0.10 and confirmation of the proportional hazards assumption by log-log plotting. To estimate relatives risks (RR) of CRS1 for each event, a variable was included to detect a potential interaction (0 = no AHF and no AKI [reference category]; 1 = AKI but no AHF; 2 = AHF but no AKI; 3 = CRS1 [AHF and AKI]). In both models the RR was adjusted by age (in years), and the categorical variables considered (as described above) were sex, LVD, acute myocardial infarction with ST segment elevation (AMI-ST), diabetes mellitus, anemia, CKD, dyslipidemia, smoking, obesity, excessive alcohol consumption, PVD, COPD, previous coronary artery disease, arterial hypertension, elevated troponin, and revascularization (no revascularization as the reference). Later, two more models were built to adjust the RR associated with CRS1 severity for the same variables, with no CRS1 as the reference category.

Secondarily, we also carried out sensitivity analyses by restricting our study cohort to determine whether the association of CRS1 with in-hospital mortality persisted in: (1) patients with short hospitalization, excluding those hospitalized longer than 1 week, (2) patients with long hospitalization, excluding those hospitalized for 1 week or less, and (3) patients stratified by age at admission (≤65 and >65 years). All analyses were done with the IBM SPSS Statistics 21 software package.

## Results

The study group included a total of 1912 incident cases of ACS. Of these patients, 113 (5.9%) died during the index hospitalization, and 396 (20.7%) were readmitted or died during the first year after discharge. On average women (n = 552) were 5 years older than men, and they had a higher frequency of CRS1 (14.5% versus 10.0%; p = 0.005).

Table 1 shows that CRS1 was associated with older age and shorter time to death or readmission (p = 0.011). The prevalence of CKD, anemia and PVD and the incidence of LVD were higher in patients with CRS1 (p<0.001). The incidence of CRS1 per 1000 person-days of hospital stay was higher in women (11.2 [95% CI = 9.3–14.5]) than in men (8.2 [95% CI = 6.8–9.7]; p = 0.012). More than half of the patients (56.6% [95% CI = 47.4–65.6]) who died during their hospital stay had CRS1. The positive predictive value of CRS1 was 29.6% (95% CI = 23.9–36.0) for death, and 51.4% (95% CI = 44.8–58.0) for readmission or death after discharge.

**Table 1:** Distribution of variables in patients with ACS according to sex and CRS1 status.

	No CRS1 n = 1696	CRS1 n = 216	P
Age (years)	63.1±12.5	70.3±12.1	<0.001
Sex (women)	27.8 (25.7–29.9)	37.2 (30.7–43.7)	0.004
In-hospital death	2.9 (2.1–3.7)	29.6 (23.9–36.0)	<0.001
Days to in-hospital death*	8.0 (6.0–13.0)	10.0 (6.0–19.0)	<0.001
Death or readmission after discharge	16.8 (15.0–18.6)	51.4 (44.8–58.0)	<0.001
Days to death or readmission *	93.0 (30.0, 189.0)	41.5 (14.8, 93.5)	0.011
Current infarction with ST segment elevation	39.7 (37.4–42.0)	49.8 (43.1–56.5)	0.005
Left ventricular dysfunction	15.7 (14.0–17.4)	47.5 (40.8–54.2)	<0.001
Cardiogenic shock	1.2 (0.7–1.7)	29.6 (23.9–36.0)	<0.001
Troponin elevation	72.1 (70.0–74.2)	94.4 (91.3–97.5)	<0.001
Chronic kidney disease	8.7 (7.4–10.0)	32.6 (26.3–38.9)	<0.001
Smoker	36.7 (34.4–39.0)	27.0 (21.1–32.9)	<0.001
Ex-smoker	24.6 (22.6–26.7)	19.5 (14.2–24.8)	
Excess alcohol consumption	11.8 (10.3–13.3)	11.2 (7.0–15.4)	0.910
Obesity	34.9 (32.6–379.2)	28.3 (22.3–34.3)	0.594
Hypertension	62.5(60.2–64.8)	70.7 (64.6–76.8)	0.019
Type 2 diabetes	32.8 (30.6–359.0)	53.5 (46.8–60.2)	<0.001
Anemia	20.0 (18.1–21.9)	47.9 (41.2–54.6)	<0.001
Dyslipidemia	51.5 (49.1–53.9)	51.2 (44.5–57.9)	0.931
Peripheral vascular disease	7.1 (5.9–8.3)	14.0 (9.4–18.6)	<0.001
Chronic obstructive pulmonary disease	4.4 (3.4–5.4)	9.3 (5.4–13.2)	0.002
No revascularization	27.3 (25.2–29.4)	38.2 (31.7–44.7)	0.004
Percutaneous revascularization	55.8 (53.4–58.2)	45.9 (39.3–52.3)	
Surgical revascularization	16.9 (15.1–18.7)	15.9 (11.0–20.8)	
Angiography	84.4 (82.7–86.1)	75.5 (69.8–81.2)	0.001
No revascularization after angiography	15.8 (14.1–17.5)	20.5 (15.1–25.8)	0.257
Previous coronary disease (AMI with ST)	10.5 (9.0–12.0)	11.6 (7.3–15.9)	0.254
Previous coronary disease (AMI without ST)	5.8 (4.7–6.9)	6.5 (3.2–9.8)	
Previous coronary disease (Stable angina)	2.2 (1.5–2.9)	4.7 (1.9–7.5)	
Previous coronary disease (Unstable angina)	5.8 (4.7–6.9)	4.2 (1.5–6.9)	

Categorical variables: % (95% CI). Age: mean ± SD.

*Time variables: median (P_25_-P_75_).

Table 2 shows that the incidence rate of in-hospital death was higher in patients with CRS1 than in patients with AHF (p = 0.030) or AKI (p<0.001) alone. When the event studied was death or readmission after discharge, the incidence rate for CRS1 was significantly higher than for AKI (p<0.001). Furthermore, the incidence of in-hospital death became higher as the severity of CRS1 increased (Table 3).

**Table 2:** Incidence rates of the two events studied here among patients with CRS1, AHF and AKI. Comparison of rates (RR, 95% CI; p).

**Event: Death during hospital stay**				
	No. events / Person-days to event	Incidence rate per 10^3^ (95% CI)	RR *CRS/AHF*	RR *CRS/AKI*
CRS1	64/2943	21.8 (16.8–27.8)	1.8 (1.0–3.4) p = 0.030	8.5 (5.4–3.6) p<0.001
AHF	14/1173	11.9 (6.5–20.0)		
AKI	25/9714	2.6 (1.7–3.8)		
**Event: Death or readmission during the first year after discharge**				
	No. events / Person-days to event	Incidence rate per 10^3^ (95% CI)	RR *CRS/AHF*	RR *CRS/AKI*
CRS1	50/37043	1.4 (1.0–1.8)	1.3 (0.8–2.4) p = 0.295	2.4 (1.7–2.3) p<0.001
AHF	17/16911	1.0 (0.6–1.6)		
AKI	122/214485	0.6 (0.5–0.7)		

**Table 3:** Incidence rates according to the severity of CRS1. Comparison of rates (RR, 95% CI; p).

**Event: Death during hospital stay**				
	No. events / Person-days to event	Incidence rate per 10^3^ (95% CI)	RR	p
No CRS	49 /20446	2.4 (1.8–3.2)	1	-
CRS1 severity 1	0/391	0.0 (0.0–9.4)	0 (0.0–3.3)	0.395
CRS1 severity 2	7/549	12.8 (5.1–26.3)	5.3 (2.2–11.2)	<0.001
CRS1 severity 3	7/478	14.6 (5.9–30.1)	6.1 (2.6–12.9)	<0.001
CRS1 severity 4	50/1525	32.8 (24.3–43.2)	13.7 (9.2–20.3)	<0.001
**Event: Death or readmission during the first year after discharge**				
	No. events / Person-days to event	Incidence rate per 10^3^ (95% CI)	RR	p
No CRS	285/511184	5.6 (4.9–6.3)	1	-
CRS1 severity 1	8/7925	10.1 (4.3–19.9)	1.8 (0.9–3.7)	0.090
CRS1 severity 2	17/8757	19.4 (11.3–31.1)	3.5 (2.1–5.7)	<0.001
CRS1 severity 3	18/6653	27.1 (16.0–42.8)	4.9 (3.0–7.8)	<0.001
CRS1 severity 4	68/14177	48.0 (37.3–60.8)	8.6 (6.6–11.2)	<0.001

**Fig 1:**
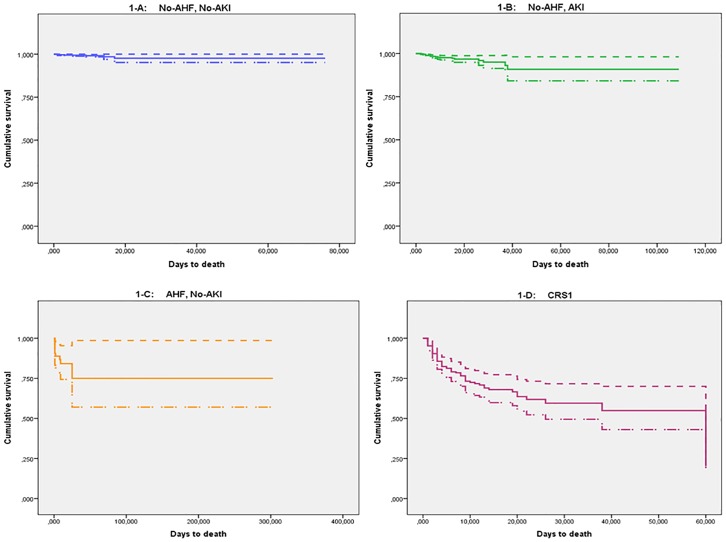
Survival curves, including confidence bands, for death during hospitalization for acute coronary syndrome in model A (Table 4). Fig 1A: No AHF and no AKI (reference category); Fig 1B: AKI but no AHF; Fig 1C: AHF but no AKI; Fig 1D = CRS1.

The multivariate Cox models found that the risk of in-hospital death in patients with CRS1 after adjustment for all other variables was greater than the sum of the effects of each component separately (Table 4, model A, RR = 18.3); for this Cox model, the linear hypothesis of no difference among the regression coefficients of CRS1 and the sum of the AHF+AKI coefficients was tested, obtaining a difference of 1.357 (p<0.001). Multivariate analysis also confirmed the direct association between the severity of CRS1 and the risk of events. The RR for in-hospital mortality increased up to 10.6 for the highest level of severity (Table 4, model C), and the highest RR for readmission or death after discharge was 4.7. Fig 1 illustrates the survival function based on model A for death during hospitalization (Fig 1A: No AHF and no AKI; Fig 1B: AKI but no AHF; Fig 1C: AHF but no AKI; Fig 1D = CRS1).

**Table 4:** Models A and B adjust the associated risk of CRS1 for the two events studied. Models C and D adjust the severity of CRS1. Only the variables that remained in each model are shown.

**Model A. Dependent variable: Time to in-hospital death**	RR	95% CI		p
CRS1 (heart failure + acute kidney injury)	18.3	6.3	53.2	<0.001
Acute heart failure only (without acute kidney injury)	7.6	1.8	31.8	0.006
AMI-ST	3.8	1.9	7.3	<0.001
Acute kidney injury only (without acute heart failure)	2.8	0.9	8.8	0.076
COPD	2.6	1.2	5.6	0.017
Left ventricular dysfunction	2.1	1.2	3.6	0.012
Age (years)	1.0	1.0	1.1	0.056
Percutaneous revascularization	0.5	0.3	1.0	0.034
Obesity	0.3	0.1	0.8	0.017
Surgical revascularization	0.2	0.1	0.7	0.009
**Model B. Dependent variable: Time to death after discharge or readmission**	RR	95% CI		p
CRS1 (heart failure + acute kidney injury)	2.5	1.7	3.8	<0.001
Acute heart failure only (without acute kidney injury)	2.3	1.2	4.3	0.008
Diabetes	1.7	1.3	2.2	<0.001
Anemia	1.7	1.3	2.2	<0.001
Acute kidney injury only (without acute heart failure)	1.4	1.0	1.8	0.043
Sex (men)	1.4	1.0	1.9	0.032
Previous coronary artery disease	1.3	1.0	1.8	0.041
Age (years)	1.0	1.0	1.0	0.089
Percutaneous revascularization	0.6	0.5	0.9	0.005
Surgical revascularization	0.6	0.4	0.9	0.011
**Model C. Dependent variable: Time to in-hospital death**	RR	95% CI		p
CRS1 severity 4	10.6	6.2	18.1	<0.001
CRS1 severity 3	7.8	3.1	19.9	<0.001
CRS1 severity 2	4.8	1.6	14.6	0.006
AMI-ST	4.0	2.2	7.3	<0.001
Peripheral vascular disease	3.0	1.5	5.9	0.002
COPD	2.0	0.1	4.1	0.070
Age (years)	1.0	1.0	1.1	0.003
Hypertension	0.6	0.4	1.0	0.053
Percutaneous revascularization	0.6	0.4	1.1	0.087
Smoking	0.4	0.2	0.7	0.002
Obesidad	0.4	0.2	0.8	0.009
Surgical revascularization	0.2	0.1	0.6	0.004
CRS1 severity 1	0	0	1.9^247^	0.969
**Model D. Dependent variable: Time to death after discharge or readmission**	RR	95% CI		p
CRS1 severity 4	4.7	3.4	6.5	<0.001
CRS1 severity 3	3.4	2.0	5.9	<0.001
CRS1 severity 2	2.5	1.4	4.5	0.002
Peripheral vascular disease	1.8	1.3	2.6	0.001
AMI-ST	1.6	1.2	2.0	<0.001
Diabetes	1.4	1.1	1.8	0.003
Anemia	1.3	1.0	1.7	0.031
Previous coronary artery disease	1.3	1.0	1.6	0.058
Age (years)	1.0	1.0	1.0	<0.001
CRS1 severity 1	0.8	0.3	2.3	0.744
Percutaneous revascularization	0.6	0.5	0.8	<0.001
Surgical revascularization	0.5	0.4	0.7	<0.001

In the sensitivity analysis the strong association of CRS1 with in-hospital mortality persisted, and was greater than the sum of the effects of each component separately in multivariate Cox models for: 1) patients with short hospitalization (n = 798; RR = 29.7 [95% CI = 6.3–140.5]; p<0.001), 2) patients with long hospitalization (n = 1114; RR = 15.5 [95% CI = 3.5–69.3]; p<0.001), 3) patients ≤65 years old on admission (n = 1006; RR = 37.8 [95% CI = 4.7–326.7]; p = 0.001), and 4) patients >65 years old on admission (n = 906; RR = 24.5 [95% CI = 5.8–103.9]; p<0.001).

## Discussion

The effect of CRS1 on mortality during the hospital stay was greater than the sum of the effects of the components of this syndrome separately, and the severity of CRS1 was an important factor in the mortality. The incidence of CRS1 was approximately 10 cases per 1000 person-days of hospital stay for ACS, but this incidence accounted for more than half of all mortality. The positive predictive value of CRS1 for death approached 30% during the hospital stay and surpassed 50% in patients who were readmitted or died from cardiovascular causes during the first year after discharge.

Both heart failure and AKI are known to be important factors in the clinical course of ACS [12]. However, as in metabolic syndrome [13], the question arises as to whether the risk contributed by CRS differs from the risk estimated as the sum of the risks of each component separately. Our study provides an answer to this question: we calculated the independent effect of CRS1 per se and of each of its components, and found that the RR for in-hospital death from CRS1 was much greater than the sum of the risks contributed by either AHF or AKI alone. We found no previous studies that categorized the severity of CRS1 to analyze its importance in mortality; although there is no standard categorization, we have devised one to show that a combination of increasingly severe impairment in heart and kidney function is associated with increasing risk of events. The modification in clinical course detected in our analysis may thus reflect unique features of the pathophysiology of this entity rather than the combination of the independent effects of each of its components. A number of pathophysiological relationships have been described between CRS and ACS. Examples of potential connections include the acute hemodynamic effects of myocardial infarction [12], the toxic effects of iodinated contrast media used for coronary angiography [14^]^ and drugs that modify the renin-angiotensin-aldosterone system [15–16]. Regarding the possible role of contrast-induced nephrotoxicity after angiography, in most patients with ACS hemodynamic deterioration is itself largely responsible for the development of CRS [17]; in fact, the proportion of patients who underwent angiography in our study was higher among those who did not have SCR1. In addition, histological and functional changes in the kidney may involve macrophage infiltration and interstitial fibrosis in the cortex [18]. Permeability of the microvessel endothelium and tubule cell apoptosis were increased in the kidneys of rats in which myocardial infarction was induced [19]. Together, these findings suggest the existence of specific mechanisms that make CRS1 a unique pathophysiological entity.

In contrast to our findings for death during hospitalization for ACS, our results did not confirm a higher risk of readmission or death after discharge when CRS1 is diagnosed than when the risks of each component of this syndrome are combined. Bivariate analysis showed that the incidence rate of readmission or death was high in patients with CRS1, but the multivariate analysis yielded no significant differences between the syndrome per se and the sum of heart failure and AKI during the first year after discharge. The severity of CRS1 is also important for this event, but the results may reflect a less important role for CRS1 in patients who survive the acute phase of ACS.

The incidence of CRS1 in patients with ACS in earlier studies varied across populations and depending on the diagnostic criteria used to define AKI [2,7,20]. In the present study we used the widely known Acute Kidney Injury Network criteria [11], and found that CRS1 was predictive of death in one third of the patients. We found no previous studies that analyzed the positive predictive value of CRS1, but even discreet increases in creatinine during the hospital stay are associated with an increased estimated incidence of CRS, and have been shown to have repercussions on the prognosis [11,21].Although a period of 48 hours has been proposed to confirm the acute nature of the deterioration in renal function [22], longer periods have also been found to have prognostic value and have also been suggested as diagnostic criteria for CRS [23]. The validity of different criteria notwithstanding, international consensus has been reached on the concept of CRS, which is defined as heart and kidney disorders in which dysfunction in one organ can induce acute or chronic dysfunction in the other [2].

Aside from CRS1 and its components, the other factors that had a significant effect on in-hospital death (AMI-ST, LVD, COPD, age and revascularization) are well known. Other factors that emerged as having a significant effect on the likelihood of readmission or death were diabetes, anemia, and previous coronary disease, whose effects have also been documented. Of particular interest was the greater protection associated with bypass versus percutaneous surgery, which agrees with the results of studies comparing the two procedures [24]. Regarding obesity, its paradoxical protective effect against death during the hospital stay is known [25] and can be attributed to residual confounding or to the high incidence of ACS in patients with an excess of adipose tissue, including some with a better initial prognosis. A similar phenomenon has also been described for the paradoxical association of tobacco smoking with lower inpatient mortality in acute ischemic stroke [26].

The main limitations of our study are those related with its observational research design. The appearance and clinical course of CRS1 may have been influenced by the use of amines and other drugs that affect the renin-angiotensin system, and this is a possibility that we cannot rule out. The use of these drugs depended on the management choices made for each individual patient by his or her doctor. Our data may be missing some baseline clinical variables and in-hospital complications, so we cannot completely exclude the possibility that unmeasured or residual confounding may explain some of our findings. It should also be noted that our findings may not be generalizable to other samples of patients in whom CRS was defined on the basis of different criteria. Specifically, we defined cases of AKI using only serum creatinine, but the criteria changed after our patients were recruited [27]. As a strength of our study, we note that the size of our cohort and our prospective follow-up design made it possible to study a large number of cases of CRS1 and final events.

We conclude that during the index hospital stay, the effect of CRS1 on mortality is greater than the sum of the effects associated to each component of this syndrome separately, and increasing severity of CRS1 worsens the prognosis. The incidence of CRS1 is approximately 10 cases per 1000 person-days of hospital stay among patients admitted because of ACS, but this incidence accounts for more than half of the mortality. The positive predictive value of CRS1 approached 30% for in-hospital death and was slightly higher than 51% after discharge.

## Supporting Information

S1 Materials Database (XLS) with information of enrolled 1912 patients. (XLS)
